# Higher T-bet and IFN-γ expression in advanced chagasic megaesophagus indicates Th1 response in the chronic phase

**DOI:** 10.1590/S1678-9946202567004

**Published:** 2025-02-03

**Authors:** Betânia Maria Ribeiro, Fernanda Rodrigues Helmo, Denise Bertulucci Rocha Rodrigues, Marcos Vinícius da Silva, Virmondes Rodrigues

**Affiliations:** 1 Universidade Federal do Triângulo Mineiro, Instituto de Ciências Biológicas e Naturais, Laboratório de Imunologia, Uberaba, Minas Gerais, Brazil; 2 Universidade Federal do Triângulo Mineiro, Centro de Educação Profissional, Uberaba, Minas Gerais, Brazil; 3 Universidade de Uberaba, Laboratório de Biopatologia e Biologia Molecular, Uberaba, Minas Gerais, Brazil; 4 Universidade Federal do Triângulo Mineiro, Instituto de Ciências Biológicas e Naturais, Disciplina de Parasitologia, Uberaba, Minas Gerais, Brazil; 5 Instituto Oswaldo Cruz, Instituto Nacional de Ciência e Tecnologia, Neuroimunomodulação, Rio de Janeiro, Rio de Janeiro, Brazil

**Keywords:** Chagas disease, Megaesophagus, T-bet transcription factor, Gamma interferon activation factor

## Abstract

Myenteric plexus injury is responsible for the morpho-functional alterations observed in chagasic megaesophagus (CME). The inflammatory response, characterized by elevated synthesis of *IFN-*γ, *TNF-*α, and *IL-4*, contributes to the persistence of parasitism and inflammation. This study assessed the mRNA expression of cytokines, transcription factors, and metalloproteases in subjects with CME. From 2011 to 2017, esophageal samples were collected from 54 subjects with CME (38 advanced and 16 nonadvanced) and eight subjects with idiopathic megaesophagus (IME). The quantitative mRNA expression of *TNF-*α, *IFN-*γ, *IL-4*, *IL-10*, *IL-17*, *IL-22*, *IL-2*3, *IL-2*7, *T-bet*, *ROR-*γ*T*, *GATA-3*, *MMP-1*, *MMP-2*, and *TIMP-3* genes was analyzed using SYBR Green systems. *T-bet* expression was significantly higher in the CME group compared to the IME group and the *GATA-3* and *ROR-*γ*T* expression in the CME group, corroborating the higher *IFN-*γ expression observed in subjects with advanced CME. The increased *T-bet* and *IFN-*γ expression in advanced CME reflects the maintenance of a Th1 response *in situ* and the morpho-functional changes seen in the organ.

## INTRODUCTION

Chagas disease, caused by the intracellular protozoan *Trypanosoma cruzi*, is a neglected tropical disease affecting 8 to 12 million people worldwide^1,2^. More than five million of these individuals live in Latin America, with 62.4% of cases in Brazil, Argentina, and Bolivia^2^.

The acute infection phase is characterized by fever, elevated parasitemia and parasitism, lymphadenopathy, hepatosplenomegaly and an intense inflammatory response^3,4^. In contrast, the chronic phase shows a significant reduction in parasites in peripheral circulation and organs, accompanied by a specific anti-*T. cruzi* immune response^1,4^.

Although most *T. cruzi-*infected individuals remain asymptomatic throughout their lives, 30–40% may develop chronic manifestations such as cardiomyopathy, megaesophagus, and chagasic megacolon after 10–20 years of infection. The cardiac form of Chagas disease affects 20–50% of this population, while the digestive form affects 5–15%, and the cardio-digestive 2–10%^5,6^. CME is characterized by impaired peristalsis and swallowing, esophageal luminal enlargement, and muscle-layer hypertrophy. Injury to neuronal cells in the myenteric plexus, resulting from the inflammatory response to smooth muscle parasitism during the acute phase, triggers morpho-functional changes^7,8^. Research indicates that CME manifests when neuronal injury affects more than 90–95% of esophageal nerve cells^9,10^. However, experimental studies suggest that intense parasitism of neuronal cells and approximately 40% neuron cell death in the myenteric plexus can occur even during the acute infection phase, contributing to the development of the chagasic megacolon. The accumulation of transudate and necrotic foci resulting from inflammation and parasitism disrupts the muscle cells organization in the circular and longitudinal layers of the organ^6^.

The local immune response is implicated in CME pathogenesis, as CD4+ T-lymphocytes, CD8+ TIA1 cytotoxic T-lymphocytes, CD20+ B-lymphocytes, CD68+ macrophages, CD57+ natural killer cells^11,12^, and a significant number of mast cells and eosinophil cells^11-13^ are present in the inflammatory infiltrate. This favors smooth muscle cells, neurons of the myenteric plexus^8,11,14,15^, and Cajal cells (important in coordinating the peristaltic movements of the digestive tract) injury and reduction^15^. In chagasic megacolon, macrophages, eosinophils, and mast cells show a significant increase in the inflammatory infiltrate and are responsible for synthesizing pro-inflammatory cytokines (e.g., *IL-1* and *TNF-*α), free radicals, and nitric oxide, which contribute to colonic lesions and fibrosis^16^.

Although the etiopathogenesis of CME is not fully understood, the digestive form is characterized by increased synthesis of *IFN-*γ and *TNF-*α, along with high levels of *IL-4,* which may favor the persistence of parasitism and the inflammatory process^4^. In Chagas disease cardiomyopathy, in which a Th1 response predominates, extensive *in situ* expression of cytokines (e.g., *IFN-*γ and *TNF-*α), chemokines (e.g., *CCL-5* and *CXCL9*), and chemokine receptors (e.g., *CCR5* and *CXCR3*) by myocardial mononuclear infiltrate cells contributes to increased T-lymphocyte chemotaxis and heightened activation, extent, and intensity of myocarditis^17,18^, as well as alterations in cardiac electrical impulse conduction^19^. *IFN-*γ, which is essential for effective trypanocidal action during the acute infection phase, is also associated with increased TCD8+ cytolytic activity and severe cardiac muscle cells damage in the chronic phase^20^.

Therefore, this study aims to evaluate the mRNA expression of *TNF-*α, *IFN-*γ, *IL-4*, *IL-10*, *IL-17*, *IL-22*, *IL-23*, *IL-27*, *T-bet*, *ROR-*γ*T*, *GATA-3*, *MMP-1*, *MMP-2*, and *TIMP-3* in esophageal fragments obtained from individuals with CME.

## MATERIALS AND METHODS

### Ethical aspects and characterization of individuals

Individuals diagnosed with megaesophagus, who were part of the Digestive Tract Surgery Outpatient Service of the Federal University of Triangulo Mineiro (UFTM) from 2011 to 2017, participated in this research. Serological tests for Chagas disease, contrast-enhanced esophageal X-rays, manometry, and fragments from the lower third of the esophageal sphincter (cardia) were collected from all participants following esophagectomy. Two groups were formed: chagasic megaesophagus (CME) and idiopathic megaesophagus (IME). Based on the degree of esophageal impairment, CME cases were classified as advanced (characterized by dolicho-megaesophagus with significant contrast retention and a progressive reduction in the contraction pressure of the esophageal body) or nonadvanced (esophagus with a diameter of up to 4 cm, potentially showing slight contrast retention while maintaining average contraction pressure). This classification was established by the digestive surgery team based on clinical evaluation, X-ray imaging, and esophageal manometry^21^. The UFTM Research Ethics Committee (protocol Nº 1307) approved this research, and all participants signed a consent form after receiving clarification. All procedures adhered to the ethical standards of the committee and the 1975Helsinki Declaration, revised in 1983.

Initially, 62 individuals were evaluated, of whom 54 were diagnosed with CME (33 men and 21 women, mean age 66 years [66.1 ± 12.6]), all from the Triangulo Mineiro region. Eight individuals were diagnosed with IME (four men and four women, mean age 36 years [36.0 ± 16.2]. Thirty-eight had advanced CME, while 16 had nonadvanced CME based on the classification criteria established by esophageal radiological evaluation and manometry^21^.

### RNA extraction

Total RNA extraction was performed using an RNA extraction kit (RNA SV Total RNA Isolation System^®^, Promega, Madison, WI, USA), following the manufacturer’s recommendations. Fragments stored in liquid nitrogen were thawed at room temperature and mechanically macerated. The resulting material was transferred to microtubes containing RNA lysis buffer and mixed in a vortex. Afterwards, 350 μL of RNA dilution buffer was added to this solution. The samples were heated to 70 °C, for 3 min and centrifuged at 13,000×g at 4 °C, for 3 min. The supernatant was transferred to new microtubes containing a separation membrane, and 200 µL of 95% alcohol was added before centrifugation at 13,000×g at 4 °C, for 1 min. The eluate was discarded, and 600 μL of RNA wash solution was added, followed by another centrifugation at 13,000×g at 4 °C, for 1 min. The eluate was discarded, and to eliminate contaminating DNA, 50μL of DNase incubation mix was added for 15 min at room temperature. The membrane containing RNA was then transferred to another microtube, and 30 μL of nuclease-free water was added before centrifugation at 13,000×g at 4 °C, for 1 min. The RNA obtained was eluted in 30 μL of RNase-free water and quantified using the NanoDrop^®^ 2000 spectrophotometer (Thermo Fisher Scientific, Inc., Waltham, MA, USA) for complementary DNA (cDNA) synthesis.

### cDNA synthesis

To synthesize cDNA, 16µL of RNA and 4µL of Random Prime buffer were incubated in the MJ Research PTC-100 thermocycler (Bio-Rad Laboratories, Inc., Hercules, CA, USA) at 70 °C for five min. After cooling, a solution containing 6 μL of deionized water, 8 μL of 5x buffer, 2.4 μL of MgCl2, 1.6 μL of deoxynucleotide triphosphates (2.5 mM), and 2 μL of Moloney Murine Leukemia Virus Reverse Transcriptase (GoScriptTM Reverse Trancription System^®^, Promega) was added. The solution was subjected to a thermal cycler for a 15-min cycle at 25 °C and another for 90 min at 42 °C. Finally, each sample had 40 μL of cDNA quantified using the NanoDrop^®^ ND2000 and subsequently stored at −20 °C for reverse transcription-quantitative polymerase chain reaction (RT-qPCR).

### RT-qPCR reaction

Relative mRNA expression analysis was conducted on genes including *TNF-*α, *IFN-*γ, *IL-4*, *IL-10*, *IL-17*, *IL-22*, *IL-23*, *IL-27*, *T-bet*, *ROR-*γ*T*, *GATA-3*, *MMP-1*, *MMP-2*, and *TIMP-3* in cDNA samples using the SYBR Green system in the GeneAmp 7000 device (Applied Biosystems, Foster City, CA, USA; Table 1). The reaction was performed using the FastStart Universal SYBR Green Master kit (Roche Diagnostics Corporation, Indianapolis, IN, USA) according to the manufacturer’s recommendations. For each sample, 6.5μL of the SYBR Green mix, 0.5μL of sense and anti-sense primers, 2.5μL of cDNA, and 5μL of sterile water were used. The amplification reaction consisted of an incubation phase at 50 °C for two min and at 95 °C for 10 min to inactivate transcriptase, followed by 40 cycles at 95 °C for 15 s, 58 °C for 30 s, and 72 °C for 30 s to obtain the dissociation curve used for amplification specificity analysis. The results were analyzed based on the cycle threshold value, which corresponds to the number of cycles in which amplification reached the threshold. The arithmetic equation for obtaining relative DNA quantification was QR = 2^-^, according to the Applied Biosystems published bulletin (user manual Nº 2: Relative Quantification of Gene Expression – PN: 4303859). Table 1 describes the primer sequences used; the primers were designed using the OligoPerfect™ Designer program (Invitrogen, Thermo Fisher Scientific) and synthesized by Exxtend.

**Table 1:** Primer sequences used in the RT-qPCR reaction.

Gene	Sequence (5’ 3’)
Hu β-actin	F - TGA CTC AGG ATT TAA AAA CTG GAA AF - GCC ACA TTG TGA ACT TTG GG
Hu *IFN-*γ	F - GAG AAC CCA AAA CGA TGC A AF - ACT TCT TTG GCT TAA TTC TCT CG
Hu *IL-4*	F - GAA GGA AGC CAA CCA GAG TA AF - GAT CGT CTT TAG CCT TTC CA
Hu *IL-10*	F - TTC CCT GAC CTC CCT CTA ATT AF - GCT CCC TGG TTT CTC TTC CTA A
Hu *IL-17*	F - TTT CTC CCC TAG ACT CAG GCT T AF - GTT GGT CTG TTG ATC TGT GAG G
Hu *IL-21*	F - CCA CAA ATG CAG GGA GAA GA AF - GAA TCA CAT GAA GGG CAT GTT
Hu *IL-22*	F – CAATTAGATGCCCCAAAGCG AF - TGGAGTTTGGCTTCCCATCTT
Hu *IL-23*	F - CAG ATT TGA GAA GAA GGC AAA AA AF - GCA GCA ACA GCA GCA TTA CA
HU *IL-27*	F - CCA GTA ACT GAA AGC CCC TCT AF - AAC CAT CAT CTC CCT AAA CAA TAA A
Hu *T-bet*	F - ATTGTGCTCCAGTCCCTC CAT AF - TCACCTCAACGATATGCAGCC
Hu *GATA-3*	F – GGCGCCGTCTTGATACTTTCA AF - AGATTGCGTTGCTCGCTCTGT
Hu *ROR-*γ*T*	F - TCA GGT TTG CTG TTC CTA CA AF - GTT CTT GTG AGC TGT GTT GC
Hu *TNF-*α	F - TTC TGG CTC AAA AAG AGA ATT G AF - TGG TGG TCT TGT TGC TTA AAG
Hu *MMP-1*	F - GAT TGA AAA TTA CAC GCC AGA T AF - TCT CAA TGG CAT GGT CCA
Hu *MMP-2*	F - ACT GTT GGT GGG AAC TCA GA AF - TTG TTG CCC AGG AAA GTG
Hu *TIMP-3*	F - TTC TCA GCG AGG ATG GCA CTT AF - AAA CAC GGT TCA GGA TGC TGG

F = sense; AF = anti-sense.

### Statistical analysis

Data were analyzed using the GraphPad Prism^®^ software (version 7.0, GraphPad, Inc., La Jolla, CA, USA). The variables were subjected to Shapiro-Wilk’s normality test and the *F*-test to compare the coefficients of variance. Mann-Whitney’s (U) test was applied to compare both groups, and Wilcoxon’s test was employed for the analysis of repeated measures. Results were considered significant at *p* < 0.05.

## RESULTS

Gene expression analysis of T-bet, GATA-3, ROR-γT, MMP-1, MMP-2, TIMP-3, IFN-γ, TNF-α, IL-4, IL-10, IL-17, IL-22, IL-23, and IL-27 in individuals with CME and IME

**Figure 1:**
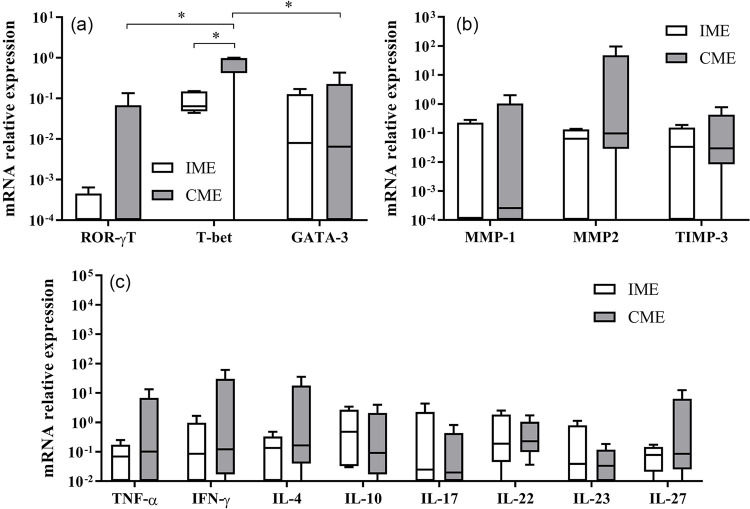
mRNA relative expression of ROR-γT, T-bet, GATA-3, MMP-1, MMP-2, TIMP-3, IFN-γ, TNF-α, IL-4, IL-10, IL-17, IL-22, IL-23, and IL-27 in individuals with idiopathic megaesophagus (IME) and chagasic megaesophagus (CME). Horizontal lines represent median values, boxes represent the 25th to 75th percentiles, and vertical lines represent the 10th to 90th percentiles. Within every panel, * represents statistically significant differences (p < 0.05). Relative DNA quantification was estimated using the equation QR = 2-ΔΔCt.

The *T-bet* transcription factor (*p* = 0.007) exhibited significantly higher expression in the CME group compared to the IME group. However, *GATA-3* and *ROR-*γ*T* expression did not significantly differ between the CME and IME groups (Table 2). When comparing *T-bet*, *GATA-3*, and *ROR-*γ*T* expression among individuals with CME, a higher *T-bet* expression was also observed in relation to *GATA-3* and *ROR-*γ*T* expression (*p* < 0.0001, Figure 1A).

**Table 2:** Difference in the relative mRNA expression of *T-bet*, *GATA-3*, *ROR-*γ*T*, MMP-1, *MMP-2*, *TIMP-3*, *IFN-*γ, *TNF-*α, *IL-4*, *IL-10*, *IL-17*, *IL-22*, *IL-23*, and *IL-27* between idiopathic megaesophagus (IME) and chagasic megaesophagus (CME) groups.

	IME	CME	*p*-value
*T-bet*	1.176 (0.284–90.359)	89.239 (0–613.364)	0.007*
*GATA-3*	0.835 (0–5.972)	0.254 (0–25.023)	0.854
*ROR-*γ*T*	0 (0–1.473)	0 (0–19.664)	0.183
*MMP-1*	0.022 (0–6.264)	0.134 (0–7.744)	0.965
*MMP-2*	0.510 (0–2.192)	1.502 (0.068–6.370)	0.266
*TIMP-3*	0.325 (0–0.912)	0.554 (0.003–4.155)	0.802
*IFN-*γ	1.446 (0–3.169)	0.975 (0–36.057)	0.635
*TNF-*α	0.693 (0–2.812)	1.053 (0–9.336)	0,300
*IL-4*	1.121 (0.427–1.547)	1.361 (0–10.63)	0.242
*IL-10*	1.462 (0–6.211)	1.891 (0–80.896)	0.290
*IL-17*	1.539 (0.317–42.754)	1.241 (0–50.022)	0.346
*IL-22*	1.923 (0–4.709)	0.830 (0–4.013)	0.842
*IL-23*	0.425 (0–2.891)	0.711 (0–3.616)	0.634
*IL-27*	0.746 (0–1.782)	0.6 (0–31.162)	0.576

*represents statistically significant differences (*p* < 0.05); relative DNA quantification was calculated using the formula QR = 2^-ΔΔCt^

The mRNA expression for *MMP-1*, *MMP-2*, *TIMP-3* (Figure 1B), *IFN-*γ, *TNF-*α, *IL-4*, *IL-10*, *IL-17*, *IL-22*, *IL-23*, and *IL-27* (Figure 1C) did not significantly differ between the CME and IME groups (Table 2).

Gene expression analysis of T-bet, GATA-3, ROR-γT, MMP-1, MMP-2, TIMP-3, IFN-γ, TNF-α, IL-4, IL-10, IL-17, IL-22, IL-23, and IL-27 in individuals with advanced and non-advanced CME

**Figure 2:**
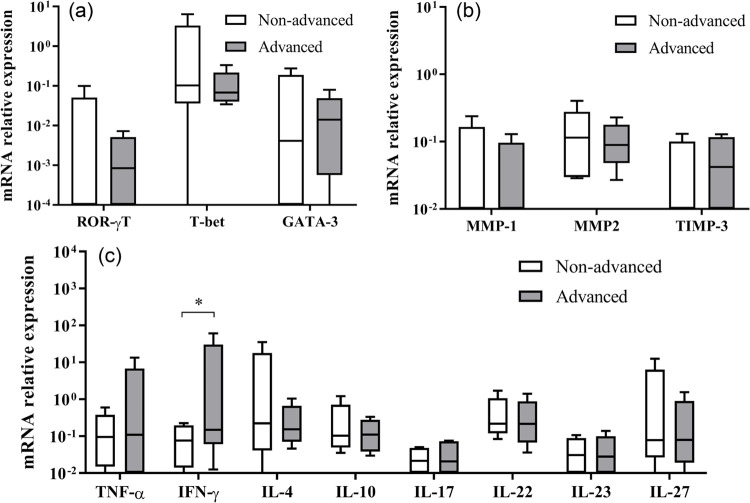
mRNA relative expression of ROR-γT, T-bet, GATA-3, MMP-1, MMP-2, TIMP-3, IFN-γ, TNF-α, IL-4, IL-10, IL-17, IL-22, IL-23, and IL-27 in individuals with nonadvanced and advanced chagasic megaesophagus. Horizontal lines represent the median values, boxes represent the 25th to 75th percentiles, and vertical lines the 10th to 90th percentiles. Within every panel, * represents statistically significant differences (p < 0.05). Relative DNA quantification was estimated using the equation QR = 2-ΔΔCt.

The expression of *T-bet*, *GATA-3,* and *ROR-yT* (Figure 2A), as well as *MMP-1*, *MMP-2,* and *TIMP-3* (Figure 2B), did not significantly differ between advanced and nonadvanced CME groups (Table 3).

**Table 3:** Difference in the relative mRNA expression of *T-bet*, *GATA-3*, *ROR-*γ*T*, MMP-1, *MMP-2*, *TIMP-3*, *IFN-*γ, *TNF-*α, *IL-4*, *IL-10*, *IL-17*, *IL-22*, *IL-23*, and *IL-27* between non-advanced and advanced chagasic megaesophagus.

	Non-advanced	Advanced	*p*-value
*T-bet*	88.839 (0–613.364)	111.746 (0–243.808)	0.266
*GATA-3*	0.114 (0–7.171)	0.469 (0–16.798)	0.977
*ROR-*γ*T*	0 (0–6.509)	0.696 (0–19.664)	0.497
*MMP-1*	0.023 (0.001–7.744)	0.029 (0.001–4.208)	0.592
*MMP-2*	1.791 (0.226–6.370)	1.514 (0.435–12.349)	0.901
*TIMP-3*	1.088 (0.002–4.155)	0.804 (0.002–2.295)	0.423
*IFN-*γ	0.686 (0–8.118)	1.298 (0.188–15.097)	0.030*
*TNF-*α	0.902 (0–2.785)	1.354 (0–9.336)	0.711
*IL-4*	1.342 (0–292.853)	1.779 (0.381–8.647)	0.529
*IL-10*	1.695 (0–24.692)	2.092 (0–7.939)	0.862
*IL-17*	1.270 (0–44.339)	1.652 (0–14.923)	0.345
*IL-22*	0.844 (0–4.013)	0.789 (0–3.291)	0.722
*IL-23*	0.797 (0–2.760)	0.711 (0–3.616)	0.629
*IL-27*	0.546 (0–4.556)	1.213 (0–31.162)	0.478

*represents statistically significant differences (*p* < 0.05); relative DNA quantification was calculated using the formula QR = 2^-ΔΔCt^

*IFN-*γ expression (*p* = 0.030) was significantly higher in the advanced CME group compared to the nonadvanced group. *TNF-*α, *IL-4*, *IL-10*, *IL-17*, *IL-22*, *IL-23*, and *IL-27* expression did not significantly differ between groups (Figure 2C and Table 3).

## DISCUSSION

The immune response in *T. cruzi* infection and its signaling pathways remain poorly understood, making it challenging to comprehend the injury process and disease progression in affected individuals. Identifying the prevalent inflammatory cells and chemical mediators *in situ* may contribute to elucidating the etiopathogenesis and understanding the characteristics of CME.

This study analyzed the mRNA expression of cytokines, transcription factors, and metalloproteases in human esophageal samples with and without CME (chronic phase). Notably, *T-bet* expression was significantly higher in the CME group than in the IME group. Additionally, *T-bet* expression was significantly higher than that of *GATA-3* and *ROR-*γ*T* in the advanced CME group*.* Corroborating this finding, *IFN-*γ expression was elevated in individuals with advanced CME compared to nonadvanced CME, suggesting a Th1 inflammatory response in CME.

Studies involving peripheral blood mononuclear cell cultures indicate increased *IFN-*γ concentrations in individuals with megaesophagus^4,22^ and lower *IL-4* synthesis^4^ in those with CME, indicating a greater Th1/Th2 response^4,22^, as observed in both experimental^23,24^ and human chagasic cardiomyopathy^25-27^. Research indicates that *T-bet* mRNA expression in the myocardium of individuals with chronic Chagas cardiomyopathy is tenfold higher compared to those with non-inflammatory cardiomyopathies. Furthermore, the relative ratio of *T-bet/GATA-3* expression is significantly elevated in the Chagas group. Additionally, *IFN-*γ expression, which is 42 times higher in chronic Chagas cardiomyopathy, shows a significant correlation with increased *T-bet* expression^25^. In *T-bet* (*Tbx2* ^-^) knockout C57BL/6 mice infected with *T. cruzi*, suppression of anti-*T. cruzi* CD8+ T cell differentiation is observed^23,24^, along with a reduced number of circulating activated CD8+ T cells (related to lower expression of chemokine receptors such as *CXCR3*)^24^, chronic neutrophilia^23^ and increased susceptibility to infection^23,24^. Note that, although CD4+ T cells maintain *IL-17* and *IFN-*γ secretion, they are resistant to *IL-2* action, which inhibits Th17 cell activity^23^, indicating that *T-bet* is essential for specific CD8+ T cell expansion^24^ and limiting Th17 response^23^.

Thus, in CME, *T-bet* expression acts as an indicator of the increase in Th1 cell numbers compared to IME. This association is likely present not only in the *in situ* immune response but also systemically, since human peripheral blood mononuclear cell cultures from chagasic cardiomyopathy subjects treated with benznidazole during the acute phase demonstrated significantly increased *IFN-*γ levels after *T. cruzi* antigen stimulation, particularly in those with a higher concentration of Th1 *T-bet* ^*+*^ *IFN-*γ^*+*^ cells (T helper cells and cytotoxic T lymphocytes). This shows a strong association with the progression or worsening of cardiac form compared to individuals with indeterminate form or healthy individuals. Furthermore, *IFN-*γ synthesis was greater than that of *IL-10*, confirming the prevalence of CD25^-^ and CD25^low^ Treg lymphocyte subpopulations in these individuals^26^.

IME is also an esophagus condition characterized by neuronal cell damage in the myenteric plexus and a significant local inflammatory infiltrate accompanied by collagen deposition. CD4+ and CD8+ T-lymphocytes, and B cells are predominant in the immune response^28,29^, especially in the advanced stages of the lesion. *IFN-*γ, *IL-22*, *IL-17*, and *IL-2* seem to contribute to the pathogenesis of the disease^30^; in these individuals, there is a prevalence of CD4+/*IFN-*γ T cells in grades II and III of achalasia^28,30^; which may explain the similar *IFN-*γ expression between CME and IME groups.

Modulation of specific receptors on *IFN-*γ-producing T cells is also significant in the chronic infection phase. Research indicates that memory TCD4+ and TCD8+ (CD45RA-) lymphocytes from chagasic cardiomyopathy subjects who synthesize *IFN-*γ show significant CD127-CD132+ expression (IL-7Rα and IL-7 γc receptors, respectively) compared to chagasic cardiomyopathy subjects whose T cells do not synthesize *IFN-*γ and uninfected individuals. However, with disease progression and severity, the amount of T CD127^+^ CD132^+^ cells considerably increases, and PD-1 expression in TCD4^+^ cells was higher^31^.

However, non-*IFN-*γ synthesizers had lower STAT-5 phosphorylation, less *IL-7* receptor functionality, and reduced CD25 regulation (mainly in those with severe heart disease) in CD4^+^ T cells, accompanied by lower Bcl-2 expression in TCD4^+^ and TCD8^+^ cells after IL-7 stimulation^31^. This suggests that *IFN-*γ synthesis by memory T cells in the chronic phase depends on the activation of other signaling pathways, regardless of the clinical stage of the disease, contributing to the maintenance of the inflammatory response, which may explain the higher *IFN-*γ expression in the advanced CME group.

Moreover, increased synthesis of *IFN-*γ and *TNF-*α can compromise mitochondrial function. Analysis of the left ventricular wall in subjects with chagasic cardiomyopathy revealed a significant increase in nitrite concentration and nitrotyrosine activity (reactive nitrogen species markers) alongside reduced mitochondrial DNA quantity compared to those with non-chagasic dilated cardiomyopathy. An adult ventricular cardiomyocyte cell line (AC-16) culture also indicated a reduced mitochondrial membrane potential following *IFN-*γ stimulation, more intense when combined with *TNF-*α (ΔΨm < 10 μm^2^)^32^.

Reactive oxygen species (ROS) synthesis by AC-16 cells increased by 43%, while adenosine triphosphate (ATP) levels decreased by 58%. The highest ROS concentration favored lipid peroxidation, reduced mitochondrial DNA synthesis, and compromised the activity of the mitochondrial oxidative phosphorylation system, affecting the structure and function of cardiomyocytes. In contrast, inhibition of *IFN-*γ*, TNF-*α*,* and cellular pathways (e.g., STAT-1/NF-kB, NOS2) was essential for membrane potential recovery and reduction of oxidative stress.^32^ Similarly, the increased *IFN-*γ synthesis in advanced CME may compromise mitochondrial function and ATP metabolism in esophageal smooth muscle cells, leading to significant cell death in *T. cruzi* infection. In summary, the higher expression of *IFN-*γ in advanced CME suggests its important role in the progression of esophageal lesions in Chagas infection.

Although no significant difference was observed between *IL-4* and *IL-10* gene expression in this study, both cytokines play crucial roles in regulating the inflammatory response in Chagas disease. Research suggests that individuals at high risk of sudden death in Chagas cardiomyopathy exhibit elevated serum levels of *IFN-*γ, *TNF-*α, *IL-6*, *IL-2*, *IL-10*, and *IL-4* compared to those at low risk. *IFN-*γ, *IL-2*, and *IL-10* are key markers associated with risk stratification^33^. In contrast, higher mRNA expression of *GATA-3*, *Foxp-3*, *IL-4*, and *IL-10* is observed in the indeterminate form compared to the cardiac form. Individuals with chronic Chagas cardiomyopathy at low stroke risk show increased expression of *GATA-3*, *Foxp-3*, and *IL-10* ^34^.

Experimental research indicates that *IL-4* knockout Balb/c mice infected with *T. cruzi* exhibit moderate inflammatory infiltrate and lower *IFN-*γ expression in cardiac tissue compared to non-infected animals even in the acute phase. Interestingly, this assay showed that lower *IFN-*γ synthesis was associated with higher *IL-10* expression, rather than lower *TNF-*α or *IL-12p70* expression^35^. It is possible to infer that, in severe forms of infection, there is reduced Th2 and Treg mediator action, leading to a worsening immune response modulation in these individuals. The prevalence of the Th1 (pro-inflammatory) response is related to extensive destruction of the myenteric plexus and progression of lesions in CME.

## CONCLUSIONS

The mechanisms and primary pathways of tissue injury in CME remain poorly studied. However, our findings indicate a predominant Th1 response, with *IFN-*γ being an essential cytokine in maintaining the pro-inflammatory response during the advanced stage of the disease. An intense and persistent cytotoxic and cytolytic activity is likely responsible for the death of smooth muscle and neuronal cells in the esophageal myenteric plexus, culminating in the morpho-functional changes observed in the chronic phase.

## References

[1] Santos E, Menezes Falcao L (2020). Chagas cardiomyopathy and heart failure: from epidemiology to treatment. Rev Port Cardiol.

[2] Perez-Molina JA, Molina I (2018). Chagas disease. Lancet.

[3] Echavarria NG, Echeverria LE, Stewart M, Gallego C, Saldarriaga C (2021). Chagas disease: chronic Chagas cardiomyopathy. Curr Probl Cardiol.

[4] Ribeiro BM, Crema E, Rodrigues V (2008). Analysis of the cellular immune response in patients with the digestive and indeterminate forms of Chagas' disease. Hum Immunol.

[5] Lidani KC, Bavia L, Ambrosio AR, Messias-Reason IJ (2017). The complement system: a prey of Trypanosoma cruzi. Front Microbiol.

[6] Ricci MF, Béla SR, Moraes MM, Bahia MT, Mazzeti AL, Oliveira AC (2020). Neuronal parasitism, early myenteric neurons depopulation and continuous axonal networking damage as underlying mechanisms of the experimental intestinal Chagas' disease. Front Cell Infect Microbiol.

[7] Köberle F (1970). The causation and importance of nervous lesions in American trypanosomiasis. Bull World Health Organ.

[8] Nascimento RD, Martins PR, Lisboa AS, Adad SJ, Silveira AB, Reis DA (2013). An imbalance between substance P and vasoactive intestinal polypeptide might contribute to the immunopathology of megaesophagus after Trypanosoma cruzi infection. Hum Pathol.

[9] Adad SJ, Andrade DC, Lopes ER, Chapadeiro E (1991). Contribuição ao estudo da anatomia patológica do megaesôfago chagásico. Rev Inst Med Trop Sao Paulo.

[10] Adad SJ, Cancado CG, Etchebehere RM, Teixeira VP, Gomes UA, Chapadeiro E (2001). Neuron count reevaluation in the myenteric plexus of chagasic megacolon after morphometric neuron analysis. Virchows Arch.

[11] Reis DA, Lemos EM, Silva GC, Adad SJ, McCurley T, Correa-Oliveira R (2001). Phenotypic characterization of the inflammatory cells in chagasic megaoesophagus. Trans R Soc Trop Med Hyg.

[12] Martins PR, Nascimento RD, Lisboa AS, Martinelli PM, Reis DA (2014). Neuroimmunopathology of Trypanosoma cruzi-induced megaoesophagus: is there a role for mast cell proteases?. Hum Immunol.

[13] Pinheiro SW, Micheletti AM, Crema VO, Cobo EC, Silva AC, Adad SJ (2008). The different concentrations of mast cells in the musculature of the esophagus and the colon. Hum Pathol.

[14] Côbo EC, Silveira TP, Micheletti AM, Crema E, Adad SJ (2012). Research on trypanosoma cruzi and analysis of inflammatory infiltrate in esophagus and colon from chronic chagasic patients with and without mega. J Trop Med.

[15] Adad SJ, Silva GB, Jammal AA (2012). The significantly reduced number of interstitial cells of Cajal in chagasic megacolon (CM) patients might contribute to the pathophysiology of CM. Virchows Arch.

[16] Silveira AB, Adad SJ, Correa-Oliveira R, Furness JB, Reis DA (2007). Morphometric study of eosinophils, mast cells, macrophages and fibrosis in the colon of chronic chagasic patients with and without megacolon. Parasitology.

[17] Nogueira LG, Santos RH, Ianni BM, Fiorelli AI, Mairena EC, Benvenuti LA (2012). Myocardial chemokine expression and intensity of myocarditis in Chagas cardiomyopathy are controlled by polymorphisms in CXCL9 and CXCL10. PLoS Negl Trop Dis.

[18] Cunha-Neto E, Chevillard C (2014). Chagas disease cardiomyopathy: immunopathology and genetics. Mediators Inflamm.

[19] Silverio JC, Pereira IR, Cipitelli MC, Vinagre NF, Rodrigues MM, Gazzinelli RT (2012). CD8+ T-cells expressing interferon gamma or perforin play antagonistic roles in heart injury in experimental Trypanosoma cruzi-elicited cardiomyopathy. PLoS Pathog.

[20] Bahia-Oliveira LM, Gomes JA, Rocha MO, Moreira MC, Lemos EM, Luz ZM (1998). IFN-gamma in human Chagas' disease: protection or pathology?. Braz J Med Biol Res.

[21] Crema E, Cruvinel LA, Werneck AM, Oliveira RM, Silva AA (2003). Correlação manométrico-radiológica e sua importância no tratamento cirúrgico do megaesôfago chagásico. Rev Soc Bras Med Trop.

[22] Crema E, Monteiro IO, Gomes MG, Silva AA, Rodrigues V (2006). Evaluation of cytokines (MIG, IFN-gamma, TNF-alpha, IL-4, IL-5, and IL-10) during the different evolutive phases of chagasic esophagopathy. Clin Immunol.

[23] Guo S, Cobb D, Smeltz RB (2009). T-bet inhibits the in vivo differentiation of parasite-specific CD4+ Th17 cells in a T cell-intrinsic manner. J Immunol.

[24] Cobb D, Guo S, Lara AM, Manque P, Buck G, Smeltz RB (2009). T-bet-dependent regulation of CD8+ T-cell expansion during experimental Trypanosoma cruzi infection. Immunology.

[25] Nogueira LG, Santos RH, Fiorelli AI, Mairena EC, Benvenuti LA, Bocchi EA (2014). Myocardial gene expression of T-bet, GATA-3, Ror-?t, FoxP3, and hallmark cytokines in chronic Chagas disease cardiomyopathy: an essentially unopposed TH1-type response. Mediators Inflamm.

[26] Llaguno M, Silva MV, Batista LR, Silva DA, Sousa RC, Resende L (2019). T-Cell immunophenotyping and cytokine production analysis in patients with Chagas disease 4 years after benznidazole treatment. Infect Immun.

[27] Rodrigues DB, Reis MA, Romano A, Pereira SA, Teixeira VP, Tostes S (2012). In situ expression of regulatory cytokines by heart inflammatory cells in Chagas' disease patients with heart failure. Clin Dev Immunol.

[28] Furuzawa-Carballeda J, Aguilar-Leon D, Gamboa-Dominguez A, Valdovinos MA, Nunez-Alvarez C, Martin-del-Campo LA (2015). Achalasia: an autoimmune inflammatory disease: a cross-sectional study. J Immunol Res.

[29] Furuzawa-Carballeda J, Torres-Landa S, Valdovinos MA, Coss-Adame E, Martín Del Campo LA, Torres-Villalobos G (2016). New insights into the pathophysiology of achalasia and implications for future treatment. World J Gastroenterol.

[30] Facco M, Brun P, Baesso I, Costantini M, Rizzetto C, Berto A (2008). T cells in the myenteric plexus of achalasia patients show a skewed TCR repertoire and react to HSV-1 antigens. Am J Gastroenterol.

[31] Natale MA, Cesar G, Alvarez MG, Eiro MD, Lococo B, Bertocchi G (2018). Trypanosoma cruzi-specific IFN-?-producing cells in chronic Chagas disease associate with a functional IL-7/IL-7R axis. PLoS Negl Trop Dis.

[32] Nunes JP, Andrieux P, Brochet P, Almeida RR, Kitano E, Honda AK (2021). Co-exposure of cardiomyocytes to IFN-? and TNF-a induces mitochondrial dysfunction and nitro-oxidative stress: implications for the pathogenesis of chronic chagas disease cardiomyopathy. Front Immunol.

[33] Rodríguez-Angulo H, Marques J, Mendoza I, Villegas M, Mijares A, Gironès N (2017). Differential cytokine profiling in Chagasic patients according to their arrhythmogenic-status. BMC Infect Dis.

[34] Guedes PM, Andrade CM, Nunes DF, Pereira NS, Queiroga TB, Machado-Coelho GL (2016). Inflammation enhances the risks of stroke and death in chronic Chagas disease patients. PLoS Negl Trop Dis.

[35] Silva MV, Almeida VL, Oliveira WD, Cascudo NC, Oliveira PG, Silva CA (2018). Upregulation of cardiac IL-10 and downregulation of IFN-? in Balb/c IL-4 -/- in ccute chagasic myocarditis due to colombian strain of Trypanosoma cruzi. Mediators Inflamm.

